# Presence of Antibodies against Bluetongue Virus (BTV) in Sheep 5 to 7.5 Years after Vaccination with Inactivated BTV-8 Vaccines

**DOI:** 10.3390/v11060533

**Published:** 2019-06-08

**Authors:** Johanna Hilke, Heinz Strobel, Soeren Woelke, Melanie Stoeter, Katja Voigt, Bernd Moeller, Max Bastian, Martin Ganter

**Affiliations:** 1 Sheep Veterinary Practice Strobel, Am Hopfenberg 8, 89352 Stoffenried, Germany; drheinzstrobel@t-online.de; 2 Clinic for Swine and Small Ruminants, Forensic Medicine and Ambulatory Service, University of Veterinary Medicine Hannover, Foundation, Bischofsholer Damm 15, 30173 Hannover, Germany; Melanie.Stoeter@tiho-hannover.de (M.S.); Martin.Ganter@tiho-hannover.de (M.G.); 3 Friedrich-Loeffler-Institute, Federal Research Institute for Animal Health, Suedufer 10, 17493 Greifswald–Riems, Germany; soeren.woelke@fli.de (S.W.); Max.Bastian@fli.de (M.B.); 4 Clinic for Ruminants with Ambulatory and Herd Health Services, Ludwig-Maximilians-University Munich, Sonnenstr. 16, 85764 Oberschleissheim, Germany; k.voigt@med.vetmed.uni-muenchen.de; 5 Friedrich-Loeffler-Institute, Institute of Farm Animal Genetics, Hoeltystr. 10, 31535 Neustadt, Germany; Bernd.Moeller@fli.de

**Keywords:** bluetongue virus, sheep, vaccination, inactivated vaccine, antibody duration, BTV-8

## Abstract

Thirty-six female sheep, previously vaccinated against Bluetongue virus serotype 8 (BTV-8) using inactivated vaccines, were included in this field study. In Germany, vaccination was compulsory in 2008 and 2009, voluntary in 2010 and early 2011, and later, was prohibited in 2011. Due to their age, eighteen sheep had been vaccinated for two or more consecutive years, while a further eighteen animals had only been vaccinated once or not at all. The sheep were blood sampled five (*n* = 31) to 7.5 years (*n* = 5) after their last vaccination. All serum samples (*n* = 36) were tested for BTV group-specific antibodies by an ELISA (IDScreen^®^ Bluetongue Competition assay, ID Vet). In five of the animals, the BTV-8 serotype-specific antibody titers were measured by serum neutralization (SN). The majority of sheep that were vaccinated annually for two or more years showed a positive ELISA (14/18 sheep) and a SN (two of two sheep) result 5 years after their last vaccination. Most of the sheep vaccinated fewer than twice showed a negative ELISA result 5 to 7.5 years after their last vaccination (13/18 animals). The three animals in this group tested by SN showed one negative and two positive results. This short communication is the first to describe the presence of BTV antibodies in sheep 5 to 7.5 years after vaccination with inactivated BTV-8 vaccines.

## 1. Introduction

Bluetongue is a notifiable disease of ruminants caused by the Bluetongue virus (BTV), an RNA-virus (genus *Orbivirus* within the family *Reoviridae*) with currently 27 known serotypes [1,2,3]. The virus is primarily transmitted by *Culicoides spp.* midges [4,5] and causes severe or even fatal disease. Sheep are the most susceptible species. Cattle were known to act as a virus reservoir without showing clinical symptoms until the BTV serotype 8 (BTV-8) epidemic in Northern Europe, when cattle were also clinically affected [6]. The disease can have a considerable economic impact due to the morbidity and mortality of livestock as well as movement restrictions and control measures [7].

When the BTV serotype 8 emerged for the first time in Northern Europe in 2006, Germany opted for a control strategy using inactivated vaccines [8]. During the vaccine licensing process, a vaccination trial was initiated in cattle and sheep, testing three different inactivated BTV-8 vaccines [9,10,11]. As these proved to be highly efficient and safe, the vaccines were initially provisionally licensed and later received a central marketing authorization by the European Medicines Agency (EMA). According to the manufacturers’ instructions, all the vaccines confer immunity for the duration of one year. Following commercial availability of these vaccines, vaccination became mandatory for all domesticated ruminants in 2008 and 2009, followed by a voluntary vaccination programme from 2010 to 2011, and then vaccination was eventually prohibited. In 2012, Germany was declared BTV-free [8]. Despite the re-emergence of BTV-8 in France in 2015 [12], and in Switzerland in 2017 [13], within close proximity to the German border, Germany maintained a disease-free status until 12 December 2018 [14], when two cattle that did not show clinical symptoms were PCR-positive for BTV-8 in a routine monitoring sample. The BTV-4 has also circulated in France since 2017 [15], and, so far, no case has been detected in Germany despite ongoing surveillance. The BTV-8 strain, currently circulating, shows less viremia, pathogenicity, and vector competence than the previous BTV-8 strain [16]. Various studies have shown the presence of BTV neutralizing antibody (nAb) in cattle for three to six years following an infection, as well as vaccination [17,18,19,20]. In sheep, nAbs are known to last for at least 2.5 years [18]. To the authors’ knowledge, there are no reports in sheep of antibody persistence beyond that time frame, which led us to undertake this field investigation.

## 2. Materials and Methods

### 2.1. Ethical Statement

For this study the procedures on animals were approved by the ethics committee of the federal state government of Upper Bavaria, Germany, for farm 1-4 (Regierung von Oberbayern, Az. 55.2-1-54-2532.0-48-2016, 19 July 2016) and the ethics committee of the Lower Saxony State Office for Consumer Protection and Food Safety, Germany, for farm 5 (Niedersächsisches Landesamt für Verbraucherschutz und Lebensmittelsicherheit, Az. 33.8-42502-05-17A211, 13 Nov 2017) and were conducted in accordance with the German animal welfare legislation and the EU Directive 2010/63/EU for animal experiments.

### 2.2. Sheep

Thirty-six female sheep, all born before March 2011 and originating from five different farms, were included in the study (Table 1). All flocks had been vaccinated annually between 2008 and 2010/11 with different inactivated BTV-8 vaccines (Table 2).

In Germany, vaccinations in sheep are usually documented based on the flock level, not individually. Therefore, annual flock vaccination records did not allow us to establish the exact age of initial vaccination for the ewe lambs that were included in the annual vaccination programmes. Therefore, we assumed ewe lambs would have been vaccinated for the first time between three and nine months of age. This leads to a degree of uncertainty as to how many vaccinations the individual sheep received during the time period of this field study (Table 1).

### 2.3. Group A: Five Years after Last Vaccination

Thirty-one sheep from four different farms, all located in the federal state of Bavaria, Southern Germany, were sampled during the summer of 2016, i.e., 5 years after their last BTV vaccination in 2011. In the rural district of farms 2 and 3, one BTV-8 PCR positive ruminant was detected in August 2008, which was the only BTV finding in this rural district ever [21]. The animals from farm 2 included in this study were all born after this case (Table 1). This flock was checked daily for clinical symptoms, however no signs of disease were ever detected. The animals of farm 3 were vaccinated three months before the mentioned case, and therefore we considered them protected if any BTV-8 had been circulating. No positive animals were found in the surrounding districts of farms 1 and 4. Thus, all flocks were not considered to be naturally infected by BTV-8.

According to the farm records, eighteen sheep in this group were vaccinated annually for at least two years, whereas thirteen sheep were only vaccinated fewer than twice.

### 2.4. Group B: 7.5 Years after Last Vaccination

Five sheep on farm 5, located in the federal state of Lower Saxony, Northern Germany, were sampled in the winter of 2017, i.e., 7.5 years after their last BTV vaccination in 2010. The region had been affected by BTV-8 in 2008, with the last positively tested animal in the respective district confirmed on 8 December 2008 [21]. As the animals in our study were born in 2010, exposure to a natural BTV-8 infection was excluded. All five animals had been vaccinated once.

Blood samples were collected from the jugular vein or the vena cava [22]. The centrifuged (2300× *g* for 5 min) and decanted serum samples were stored at −20 °C until testing.

### 2.5. ELISA

All serum samples (*n* = 36) were tested for BTV group-specific antibody activities using a commercial competitive ELISA (ID Screen^®^ Bluetongue Competition assay, IDvet, Grabels, France) in accordance with the manufacturer’s instructions at the Clinic for Swine and Small Ruminants, University of Veterinary Medicine Hannover, Germany. The results were expressed as percentage negativity (PN) as compared with the negative control. The results were classified as positive (PN ≤ 50), inconclusive (50 < PN < 60), or negative (PN ≥ 60) in accordance with the cut-offs provided by the manufacturer. This competitive ELISA was validated [23] and showed a measurement sensitivity of 100% (CI95%: 99.49–100%, *n* = 754 cattle, sheep, goats) and a measurement specificity of 100% (CI95%: 99.84–100%, n = 2461 cattle, sheep, goats) (personal communication K. Klewer-Fromentin, IDvet, Grabels, France).

### 2.6. Serum Neutralization Test (SNT)

Serotype-specific neutralizing antibody (nAb) titers against BTV-8 were measured in five serum samples. These tests were carried out at the Friedrich-Loeffler-Institute (FLI), Federal Research Institute for Animal Health, Isle of Riems, Germany.

Heat inactivated serum samples were diluted five-fold in cell medium, RPMI 1640 (Biochrom, Berlin, Germany) containing 2% foetal calf serum, l-glutamin (2 mM, biochrom) and penicillin/streptomycin (100 IU/mL, Biochrom). Subsequently, sera were serially diluted two-fold. For each dilution, 50 µL were mixed in quadruplicate with 50 µL cell medium containing 1 × 10^2^ CCID50 BTV-8. This virus was isolated by the National Bluetongue Reference Laboratory at the FLI from a German dairy cow in 2007 and since then has been maintained as a reference isolate using a seed lot system. The sera were co-incubated with virus for 3 h at 37 °C and afterwards added to near confluent layers of Madin–Darby bovine kidney (MDBK) cells in 96-well flat bottom microtiter plates. After incubation for 2–3 days at 37 °C, the monolayer was scored for cytopathic effect. Neutralizing titers were given as neutralizing dose 50 (ND_50_) per milliliter, which was calculated from the reciprocal of the highest serum dilution that caused virus neutralization in 50% of the tested quadruplicates using the method developed by Spearmann and Kaerber [24].

## 3. Results

### 3.1. Group A: Five Years after Last Vaccination

In total, 16 out of the 31 animals were ELISA and/or SNT positive five years after the last vaccination with an inactivated BTV-8 vaccine (Table 1).

Of those sheep vaccinated at least twice between 2008–2011, 14/18 were ELISA positive. Two animals in this group (ID 5151 and ID 32) were analyzed for serotype-specific nAb and were highly positive for BTV-8 (both animals with a ND_50_ of 47.6).

Of those sheep vaccinated less than twice between 2010–2011, 12/13 showed negative ELISA results. In the remaining animal (ID 1382) the ELISA result was inconclusive. Two animals in this group were tested using the SNT. One of them showed no serotype-specific nAb activity at all (ID 13). The other animal (ID 1333) was highly positive for BTV-8 (ND_50_ 56.6), while showing no pan-BTV antibody activity by ELISA.

### 3.2. Group B: 7.5 Years after Last Vaccination

Two of the five animals tested positive by ELISA 7.5 years after the last vaccination (Table 1). Another two of the five animals showed inconclusive ELISA results. The remaining ELISA-negative animal (ID 894635) was positive to BTV-8 nAb by SNT (ND_50_ 12.6).

## 4. Discussion

According to our analyses, the majority of sheep vaccinated annually for two or more consecutive years were BTV seropositive five years after their last vaccination with an inactivated BTV-8 vaccine (14 of 18 sheep). The majority of animals which had received only one vaccination showed ELISA or SNT negative results (15 of 18 sheep) five to 7.5 years after the last flock vaccination. However, 7.5 years after only one vaccination, two of five animals showed a positive ELISA and another animal (1 of 5) tested positive for the BTV-8 nAbs.

Although our study included only a small number of animals, we were able to prove a longer duration of the nAb than expected and presumed by the vaccine manufacturers. This supports other studies in cattle, which proved a BTV nAb duration after inactivated vaccines for three years [17], four years [18] and even six years [19]. To our knowledge, BTV nAb has only been shown to persist in sheep for a maximum of 2.5 years following vaccination with an inactivated vaccine [18].

Inactivated BTV vaccines are considered to protect from disease and viremia by stimulating both the humoral and cellular arms of the immune system [11,25,26,27]. Cytotoxic T-cells are assumed to be involved in cross-serotype protection [11,27,28,29]. The BTV-group-specific Abs against the inner capsid protein VP7 are detected by ELISA, whereas their role in protective immunity is unclear [30]. The serotype-specific nAbs are directed against outer capsid proteins VP2, and to a smaller extent against VP5 [25]. These immunogenic components do not necessarily correspond to each other, which was also seen in two animals in our study (ID 1333 and ID 894635) that showed a negative ELISA and a positive SNT result. The detection of viremia after virus challenge is, therefore, considered the most straight-forward and meaningful way to assess vaccine efficacy [31]. However, it was beyond the scope of this field study to perform a challenge experiment. Previous studies on persisting nAbs [17,18,19] all lack a virus challenge, but rely on studies showing that nAbs are highly correlated with protection from disease [26,31,32,33]. The small amount of SN tests was a limitation of our study. Nevertheless, in previous challenge studies, individual animals were protected while showing ELISA positive and SNT negative results [9], ELISA negative results [11] or even negative results in both tests [29,34]. Therefore, it was assumed that at least the animals that showed either ELISA or SNT positive results would be protected in the case of infection. However, further research is needed to confirm this assumption by these challenge studies.

The BTV was eliminated by a compulsory vaccination campaign in 2008 and 2009, followed by voluntary vaccinations in 2010 and 2011 [8]. It is postulated that the success of this relatively short campaign might have been supported by the long duration of protective antibodies after infection and especially after vaccination with inactivated BTV vaccines [17].

Due to the close genetic relationships between strains in 2008 and 2015, it is assumed that BTV-8 circulated at a low level in France between 2008 and 2015 [35]. Bournez et al. [36] supposed that animals were tested PCR positive when the herd immunity sank below an estimated level of 20% in cattle [36].

As it is unpredictable to what extent a circulating BTV-8 strain might become more virulent, pathogenic and/or vector competent when encountering an immunologically naive ruminant population, vaccinations are still highly recommended. According to our results, re-evaluating the current vaccination schemes might be an option, as already proposed by Ayrle et al. [19].

Economic considerations often prevent flock vaccinations against BTV. In view of the long duration of immunity observed in this current study, it should be tested whether an initial immunization consisting of two doses in the first year, followed by a single injection in the second year, would protect the flock from viremia and clinical signs for several years. By considering the age distribution within the flocks it may be possible to maintain sufficient flock protection by immunizing replacements only (two doses in the first year, followed by an additional vaccination in the second year). Revaccination of the whole flock would depend on the replacement rate. At an average replacement rate of 15.5% (10–22%) in Southern Germany [37], a vaccination coverage of between 60% and 100% would be achieved in the sixth year. In flocks with lower than a 15% replacement rate, whole-flock revaccination might be necessary after five years to ensure sufficient cover to stop virus spread, as well as viremia, and to avoid the occurrence of clinical symptoms. This could be an animal friendly and economic control strategy for flocks situated in a BTV restricted area with no need to be moved. For those flocks and individual animals which leave the BTV restricted zone, annual vaccination after the initial boosted vaccination should remain mandatory to ensure virus control.

## Figures and Tables

**Table 1:** Details on animals, history of vaccination, and results of ELISA (BTV group-specific antibodies) and serum neutralization (SN, BTV-8 serotype-specific neutralising antibodies).

ID	Date of Birth	Farm/Group ^1^	Breed	Vaccinations 2008–2011 ^2^	ELISA [P/N% ^3^]	ELISA [Status]	SN [ND_50_^4^]
11,473	22.12.2005	3/A	MLS ^5^	4	26.6	Positive	
12,793	15.12.2006	3/A	MLS	4	139.8	Negative	
12,713	15.01.2007	3/A	MLS	4	108.3	Negative	
12,313	18.01.2007	3/A	MLS	4	122.8	Negative	
13,163	16.12.2007	3/A	MLS	3–4	5.6	Positive	
13,373	16.12.2007	3/A	MLS	3–4	6.5	Positive	
13,443	16.12.2007	3/A	MLS	3–4	8.3	Positive	
5151	25.02.2008	1/A	MLS	3–4	13.3	Positive	47.6
1452	07.09.2008	2/A	MLS	2–3	7.4	Positive	
22	01.10.2008	2/A	MLS	2–3	5.9	Positive	
32	06.10.2008	2/A	MLS	2–3	5.9	Positive	47.6
5541	13.10.2008	1/A	MLS	2–3	8.9	Positive	
5711	20.10.2008	1/A	MLS	2–3	8.7	Positive	
1332	25.12.2008	2/A	MLS	2–3	8.2	Positive	
14,283	01.01.2009	3/A	MLS	2–3	6.5	Positive	
1264	11.02.2009	4/A	AS ^6^	2	8.5	Positive	
5701	09.03.2009	1/A	MLS	2	8.8	Positive	
1392	22.05.2009	2/A	MLS	2	73.7	Negative	
5613	05.01.2010	3/A	MLS	1–2	114.5	Negative	
13	07.01.2010	3/A	MLS	1–2	126.4	Negative	0.1
1462	25.07.2010	2/A	MLS	1	112.5	Negative	
1512	08.11.2010	2/A	MLS	0–1	105.4	Negative	
971	20.12.2010	1/A	MLS	1	167.8	Negative	
921	30.12.2010	1/A	MLS	1	170.5	Negative	
1443	04.01.2011	3/A	MLS	0–1	150.7	Negative	
1333	05.01.2011	3/A	MLS	0–1	125.5	Negative	56.6
11,913	15.01.2011	3/A	MLS	0–1	157.4	Negative	
1382	17.01.2011	2/A	MLS	0–1	57.4	Inconclusive	
931	19.01.2011	1/A	MLS	0–1	173.3	Negative	
1463	19.01.2011	3/A	MLS	0–1	152.6	Negative	
1852	25.02.2011	2/A	MLS	0–1	86.3	Negative	
894,565	07.01.2010	5/B	BHM ^7^	1	13.397	Positive	
894,765	18.01.2010	5/B	BHM	1	53.18	Inconclusive	
894,705	26.01.2010	5/B	BHM	1	10.956	Positive	
894,635	27.01.2010	5/B	BHM	1	110.287	Negative	12.6
894,675	27.01.2010	5/B	BHM	1	57.566	Inconclusive	

^1^ group A = 5 years and group B = 7.5 years since last vaccination, ^2^ the number of vaccinations was estimated taking into account the flock vaccination records and the date of birth of the animal, ^3^ percentage negativity, ^4^ neutralizing dose 50, ^5^ merino land sheep, ^6^ alpine sheep, ^7^ black headed mutton.

**Table 2:** Vaccines used in vaccinations 2008–2011. Bluevac^®^BTV8 (CZ Veterinaria S.A., Porrino, Spain); Bovilis^®^ BTV8 (Intervet International BV, Boxmeer, the Netherlands), BTVPUR^®^ AlSap 8 (Merial S.A.S., Lyon, France); Zulvac^®^8 Ovis (Fort Dodge Animal Health, Naarden, the Netherlands); n/k = vaccine not known.

Farm	2008	2009	2010	2011
1	June, Bluevac^®^BTV8	April, Bovilis^®^ BTV8	April, BTVPUR^®^ AlSap 8	August, Zulvac^®^8 Ovis
2	July, Bluevac^®^BTV8	June, Bovilis^®^ BTV8	May, n/k	April, n/k
3	May, Bluevac^®^BTV8	April, Bovilis^®^ BTV8	April, Bovilis^®^ BTV8	April, Zulvac^®^8 Ovis
4	January, n/k	January, BTVPUR^®^ AlSap 8	January, BTVPUR^®^ AlSap 8	January, BTVPUR^®^ AlSap 8
5	n/k	n/k	June, BTVPUR^®^ AlSap 8	No vaccinations

## References

[1] OIE-Listed Diseases 2018: OIE—World Organisation for Animal Health. http://www.oie.int/en/animal-health-in-the-world/oie-listed-diseases-2018/.

[2] Verwoerd D.W., Louw H., Oellermann R.A. (1970). Characterization of Bluetongue Virus Ribonucleic Acid. J. Virol..

[3] Belbis G., Zientara S., Bréard E., Sailleau C., Caignard G., Vitour D., Attoui H. (2017). Bluetongue Virus: From BTV-1 to BTV-27. Advances in Virus Research.

[4] Du Toit R.M. (1944). The Transmission of Blue-Tongue and Horse-Sickness by Culicoides. Onderstepoort J. Vet. Sci. Anim. Ind..

[5] Wilson A.J., Mellor P.S. (2009). Bluetongue in Europe: Past, present and future. Philos. Trans. R. Soc. B Biol. Sci..

[6] Conraths F.J., Gethmann J.M., Staubach C., Mettenleiter T.C., Beer M., Hoffmann B. (2009). Epidemiology of Bluetongue Virus Serotype 8, Germany. Emerg. Infect. Dis..

[7] Gethmann J., Probst C., Sauter-Louis C., Conraths F.J. (2015). Economic analysis of animal disease outbreaks–BSE and Bluetongue disease as examples. Berliner und Munchener Tierarztliche Wochenschrift.

[8] Baetza H.-J. (2014). Eradication of bluetongue disease in Germany by vaccination. Vet. Immunol. Immunopathol..

[9] Eschbaumer M., Hoffmann B., König P., Teifke J.P., Gethmann J.M., Conraths F.J., Probst C., Mettenleiter T.C., Beer M. (2009). Efficacy of three inactivated vaccines against bluetongue virus serotype 8 in sheep. Vaccine.

[10] Gethmann J., Hüttner K., Heyne H., Probst C., Ziller M., Beer M., Hoffmann B., Mettenleiter T.C., Conraths F.J. (2009). Comparative safety study of three inactivated BTV-8 vaccines in sheep and cattle under field conditions. Vaccine.

[11] Wäckerlin R., Eschbaumer M., König P., Hoffmann B., Beer M. (2010). Evaluation of humoral response and protective efficacy of three inactivated vaccines against bluetongue virus serotype 8 one year after vaccination of sheep and cattle. Vaccine.

[12] Sailleau C., Bréard E., Viarouge C., Vitour D., Romey A., Garnier A., Fablet A., Lowenski S., Gorna K., Caignard G. (2017). Re-Emergence of Bluetongue Virus Serotype 8 in France, 2015. Transbound. Emerg. Dis..

[13] OIE World Animal Health Information System BTV Switzerland. http://www.oie.int/wahis_2/public/wahid.php/Reviewreport/Review?page_refer=MapFullEventReport&reportid=25166.

[14] Radar_Bulletin_Deutschland-Dezember_2018_oeffentlich.pdf. https://www.openagrar.de/servlets/MCRFileNodeServlet/openagrar_derivate_00019395/Radar_Bulletin_Deutschland-Dezember_2018_oeffentlich.pdf.

[15] OIE World Animal Health Information System BTV4 France. http://www.oie.int/wahis_2/public/wahid.php/Reviewreport/Review?page_refer=MapFullEventReport&reportid=21777.

[16] Flannery J., Sanz-Bernardo B., Ashby M., Brown H., Carpenter S., Cooke L., Corla A., Frost L., Gubbins S., Hicks H. (2019). Evidence of reduced viremia, pathogenicity and vector competence in a re-emerging European strain of bluetongue virus serotype 8 in sheep. Transbound. Emerg. Dis..

[17] Oura C.A.L., Edwards L., Batten C.A. (2012). Evaluation of the humoral immune response in adult dairy cattle three years after vaccination with a bluetongue serotype 8 inactivated vaccine. Vaccine.

[18] Batten C.A., Edwards L., Oura C.A.L. (2013). Evaluation of the humoral immune responses in adult cattle and sheep, 4 and 2.5 years post-vaccination with a bluetongue serotype 8 inactivated vaccine. Vaccine.

[19] Ayrle H., Mevissen M., Kaske M., Vögtlin A., Fricker R., Hoffmann B., Büttner M., Marinovic Z., Walkenhorst M. (2018). Colostral transmission of BTV-8 antibodies from dairy cows six years after vaccination. Vaccine.

[20] Eschbaumer M., Eschweiler J., Hoffmann B. (2012). Long-term persistence of neutralising antibodies against bluetongue virus serotype 8 in naturally infected cattle. Vaccine.

[21] TSIS—TierSeuchenInformationsSystem Günzburg/Hannover. https://www.tsis.fli.de/Reports/Info_SO.aspx?ts=009&guid=e61d7ae9-20ee-457c-b78b-d852c4f476d2.

[22] Ganter M. (2001). Blood sampling from the vena cava cranialis in sheep and goat using sampling systems armed by canula. Tierärztliche Praxis Ausgabe G: Großtiere/Nutztiere.

[23] Vandenbussche F., Vanbinst T., Verheyden B., van Dessel W., Demeestere L., Houdart P., Bertels G., Praet N., Berkvens D., Mintiens K. (2008). Evaluation of antibody-ELISA and real-time RT-PCR for the diagnosis and profiling of bluetongue virus serotype 8 during the epidemic in Belgium in 2006. Vet. Microbiol..

[24] Mayr A., Bachmann P., Bibrack B., Wittmann G. (1974). Quantitative Bestimmung der Virusinfektiösität (Virustitration). Virologische Arbeitsmethoden.

[25] Schwartz-Cornil I., Mertens P.P.C., Contreras V., Hemati B., Pascale F., Bréard E., Mellor P.S., MacLachlan N.J., Zientara S. (2008). Bluetongue virus: Virology, pathogenesis and immunity. Vet. Res..

[26] Lobato Z.I.P., Coupar B.E.H., Gray C.P., Lunt R., Andrew M.E. (1997). Antibody responses and protective immunity to recombinant vaccinia virus-expressed bluetongue virus antigens. Vet. Immunol. Immunopathol..

[27] Umeshappa C.S., Singh K.P., Pandey A.B., Singh R.P., Nanjundappa R.H. (2010). Cell-mediated immune response and cross-protective efficacy of binary ethylenimine-inactivated bluetongue virus serotype-1 vaccine in sheep. Vaccine.

[28] Jeggo M.H., Wardley R.C., Brownlie J. (1985). Importance of ovine cytotoxic T cells in protection against bluetongue virus infection. Prog. Clin. Biol. Res..

[29] Breard E., Belbis G., Viarouge C., Nomikou K., Haegeman A., De Clercq K., Hudelet P., Hamers C., Moreau F., Lilin T. (2015). Evaluation of adaptive immune responses and heterologous protection induced by inactivated bluetongue virus vaccines. Vaccine.

[30] Darpel K.E., Monaghan P., Anthony S.J., Takamatsu H.-H., Mertens P.P.C. (2009). Bluetongue virus in the mammalian host and the induced immune response. Bluetongue.

[31] Savini G., MacLachlan N.J., Sánchez-Vizcaino J.-M., Zientara S. (2008). Vaccines against bluetongue in Europe. Comp. Immunol. Microbiol. Infect. Dis..

[32] Oura C.A.L., Wood J.L.N., Sanders A.J., Bin-Tarif A., Henstock M., Edwards L., Floyd T., Simmons H., Batten C.A. (2009). Seroconversion, neutralising antibodies and protection in bluetongue serotype 8 vaccinated sheep. Vaccine.

[33] Jeggo M.H., Wardley R.C., Taylor W.P. (1984). Role of neutralising antibody in passive immunity to bluetongue infection. Res. Vet. Sci..

[34] Stott J., Barber T. (1985). Osburn BI Immunologic response of sheep to inactivated and virulent bluetongue virus. Am. J. Vet. Res..

[35] Bréard E., Sailleau C., Quenault H., Lucas P., Viarouge C., Touzain F., Fablet A., Vitour D., Attoui H., Zientara S. (2016). Complete Genome Sequence of Bluetongue Virus Serotype 8, Which Reemerged in France in August 2015. Genome Announc..

[36] Bournez L., Cavalerie L., Sailleau C., Bréard E., Zanella G., Servan de Almeida R., Pedarrieu A., Garin E., Tourette I., Dion F. (2018). Estimation of French cattle herd immunity against bluetongue serotype 8 at the time of its re-emergence in 2015. BMC Vet. Res..

[37] Frohnmayer S.C. (2015). Betriebsanalyse zu Produktionskennzahlen und Lämmerverlusten in baden-württembergischen Schäfereien.

